# Modified Hofmann Articulated Spacer in the Treatment of Peri-Prosthetic Joint Infection of the Knee—Surgical Technique and Early Clinical Evaluation

**DOI:** 10.3390/jcm14217605

**Published:** 2025-10-27

**Authors:** Salvatore Risitano, Simone Sanfilippo, Beatrice Limone, Stefano Artiaco, Marianna Faggiani, Marcello Capella, Alessandro Massè

**Affiliations:** Department of Orthopaedic Surgery and Traumatology, Città Della Salute e Della Scienza Torino—CTO, 10126 Turin, Italy; simone.sanfilippo@unito.it (S.S.); limonebeatrice@gmail.com (B.L.); stefano.artiaco@unito.it (S.A.); mari.faggiani@hotmail.it (M.F.); marcello.capella84@gmail.com (M.C.); alessandro.masse@unito.it (A.M.)

**Keywords:** Hofmann spacer, articulating spacer, infection, periprosthetic joint infection, total knee arthroplasty, 1.5 stage approach, 2 stage approach, revision arthroplasty, surgical technique

## Abstract

**Background/Objectives**: The rate of periprosthetic joint infection (PJI) is expected to increase in the next years worldwide, mainly due to increasing volume of total joint replacement, longer prosthesis lifespans, and patients with multiple comorbidities. The aim of this study is to describe our personal technique, the modified Hofmann Articulated Spacer (mHAS), in which a CR femoral shield and a partially threaded cannulated screw are inserted into the liner replicating a tibial stem, and to evaluate the efficacy of the spacer as a definitive treatment option in selected patients with knee infections. **Methods**: A consecutive series of 132 patients were treated for orthopedic infection at the Orthopedic and Trauma Center, University of Turin, between November 2023 and May 2025. All patients included in the study had undergone knee prosthesis removal followed by the implantation of a modified Hofmann Articulated Spacer (mHAS). Functional recovery was evaluated through clinical examination, particularly knee range of motion, and patient-reported outcome measures (PROMs), including the Knee Society Score (KSS), Oxford Knee Score (OKS), and the EQ-5D-5L Visual Analogue Scale (VAS). **Results**: Nine patients were enrolled in the study, at a mean follow-up of 8.12 months (range: 3–13). The mean range of motion of the knee was 95 degrees (range: 80–120°, SD: 15°). The Knee Society Score (KSS) presented a mean value of 71.9 (SD: 18.11). The Oxford Knee Score (OKS) showed a mean value of 30.8 (SD: 8.5). The EuroQol-5 Dimension-5 Level Visual Analogue Scale (EQ-5D-5L VAS) scores demonstrated an excellent quality of life among the participants. **Conclusions**: The Modified Hofmann Articulated Spacer demonstrated good functional, qualitative outcomes and eradication rates in patients who underwent the first-stage revision TKA for PKI. This has led us to propose it as a definitive treatment option for more critical and low-demand patients and to postpone the second-stage surgery in the remaining cohort due to satisfactory spacer joint function without pain.

## 1. Introduction

Total knee arthroplasty (TKA) represents one of the most commonly performed and reliably successful procedures in orthopedic surgery for patients with end-stage knee osteoarthritis. Symptomatic knee osteoarthritis affects approximately 240 per 100,000 patients, with around 400,000 primary TKA surgeries carried out annually in the United States [1]. Periprosthetic joint infection (PJI) occurs in 1 to 2% of primary and in 4% of revision knee arthroplasty; its incidence is projected to rise globally due to increasing volume of total joint replacement, longer prosthesis lifespans, and patients with multiple comorbidities [2,3]. PJI classification primarily relies on the timing of symptom onset, distinguishing Acute PJI (<4 weeks after surgery or <3 weeks duration of symptoms) and Chronic PJI (>4 weeks after surgery or >3 weeks duration of symptoms) [4]. For acute infection, the treatment of choice is represented by DAIR (Debridement, Antibiotic, and Retention of the Implant) or DAPRI (Debridement, Antibiotic Pearls, and Retention of the Implant). This treatment is performed with a radical removal of necrotic or inflamed tissue, synovectomy, extensive irrigation with sterile saline, replacement of mobile prosthetic components, and placement of antibiotic pearls at the infection site [5]. In a chronic infection, treatment can follow either a one-stage or two-stage revision approach. The one-stage method involves complete removal of the infected prosthesis and implantation of a new one, commonly practiced in Europe but less frequently than two-stage procedures due to the potential for severe complications if failure occurs [6,7]. In the case of a two-stage revision, firstly the infected prosthetic implant is completely removed and antibiotic spacers are implanted (static or articulating spacer) while the revision total knee arthroplasty is performed in a second surgical procedure. Static spacers, often hand-prepared with antibiotic-loaded cement and reinforced with metal rods, create a temporary arthrodesis to maintain joint space. They significantly impact knee function and quality of life. Their use is recommended in cases with poor soft tissue condition, extensor mechanism rupture, or major bone loss [8,9]. Moreover, two types of articulating spacers could be utilized: the Mold spacer and the Hofmann spacer. Hofmann in 1995 proposed, for the first time, the metal-on-poly spacer. Incorporate a femoral component, coupled with a fresh polyethylene liner directly cemented onto the tibia [10]. This technique facilitates earlier knee motion without increasing infection risk or spacer failure [11]. Recently, a 1.5-stage approach has gained attention, especially for patients with multiple comorbidities. It involves the maintenance of an articulated spacer for an indefinite period, delaying revision until reinfection, functional decline, or aseptic loosening occurs [12,13]. The primary objective of this study was to evaluate the evolution of treatment strategies for chronic periprosthetic joint infections (PJI) of the knee using an mHAS with a specific focus on the possibility of avoiding reimplantation in patients who achieve satisfactory functional outcomes, remain infection-free, and present with significant comorbidities that may contraindicate further surgical intervention.

## 2. Materials and Methods

Approval from the Ethics Committee was obtained prior to this retrospective review. A consecutive series of 132 patients were treated for orthopedic infection at the Orthopedic and Trauma Center, University of Turin, between November 2023 and May 2025. Thirty-eight patients presented with periprosthetic infections, and twenty-two patients presented with chronic periprosthetic joint infection (PJI) of the knee, as defined by the criteria of the International Consensus Meeting and the European Bone and Joint Infection Society. Inclusion criteria were as follows: chronic periprosthetic joint infection, removal of an infected total knee prosthesis followed by implantation of an mHAS, age > 18 years, availability of complete clinical, microbiological, and radiographic data, and minimum follow-up of 3 months. Exclusion criteria were as follows: patients with acute or early PJI, infection related to fractures or septic arthritis, PJI involving hip prosthesis, patients with incomplete clinical records or loss to follow-up before the minimum follow-up period, and patients treated with static spacers or a one-stage procedure. Finally, nine patients were enrolled for inclusion in this retrospective study. All patients included in the study had undergone knee prosthesis removal followed by the implantation of a modified Hofmann Articulated Spacer (mHAS). All surgical procedures were performed by senior orthopedic surgeons using a standardized protocol. Following removal of the infected prosthesis, a custom-made articulated antibiotic-loaded cement spacer (mHAS) was implanted. The mHAS used in this study is characterized by a cemented femoral shield, a polyethylene liner, and an AO screw, which replaces the tibial component. The spacer is further enhanced with antibiotic-loaded cement, ensuring the release of antibiotics to manage infection during the treatment period. Postoperative management included weight-bearing as tolerated with the aid of crutches or a walker and initiation of physiotherapy with knee mobilization within a controlled range of 0–90°. Complete demographic data were collected for all patients (age, sex, and relevant comorbidities), including microbiological characteristics of the infecting organism. Additional information regarding the type of primary implant, previous surgical history, and time elapsed since the index arthroplasty was also documented to ensure comprehensive clinical profiling. Patients were monitored through scheduled follow-up visits. Infection status was assessed through serial laboratory testing of inflammatory markers. Infection eradication requires the absence of clinical signs of infection, normalization of CRP and ESR levels, and negative intraoperative cultures at reimplantation. Functional recovery was evaluated through clinical examination, particularly the knee range of motion. Patient-reported outcome measures (PROMs), including the Knee Society Score (KSS), Oxford Knee Score (OKS), and the EQ-5D-5L Visual Analogue Scale (VAS), were collected preoperatively and at the end of the follow-up period.

### Surgical Technique

**Figure 1:**
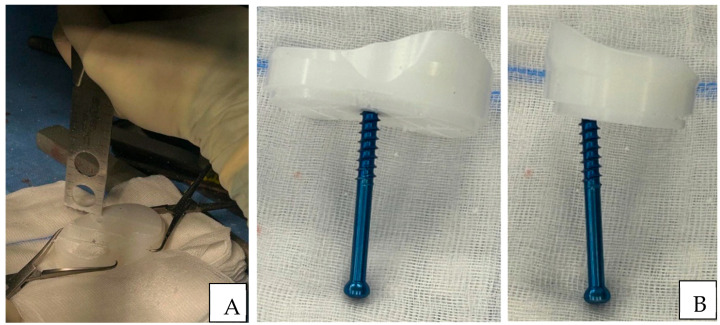
Preparation of tibial trial liner. (**A**) Using an oscillating saw, multiple cuts are made on the inferior surface of the polyethylene liner to promote better interdigitation of the cement and enhance overall fixation. (**B**) Anteroposterior and lateral views of a 6.5 mm diameter partially threaded cannulated screw inserted into the liner with a distal-to-proximal orientation, replicating the function of a tibial stem.

The patient was placed supine on the surgical table with a tourniquet applied to the thigh of the operated limb. A lateral post, proximal to the thigh, at the level of the tourniquet, and a fixed roller under the foot were placed to keep the knee at a 90° flexion and allow hyperflexion during surgery. The previous skin incision was generally used as a surgical approach. Before opening the capsule and accessing the joint, an arthrocentesis was performed to obtain a synovial fluid sample for chemical-physical examinations. The surgical approach involved a medial parapatellar exposure to access the implant. Complete and meticulous removal of the synovial tissue was carried out with careful attention to preserving the continuity of the quadriceps and patellar tendons. Samples of synovial tissue were sent for culture examination. Removal of all fibrous scar tissue in proximity to the prosthetic implant was performed. The prosthetic implant was removed in the following sequence: polyethylene, femoral, and tibial components. The removed implant was sent for sonication. Polyethylene was leveraged and lifted upwards using a small osteotome or a pointed Hohmann retractor under the liner. In an attempt to minimize bone loss, the femoral component was detached from the bone surface using a reciprocating saw blade and chisel until complete mobilization and then hammered until complete removal. A reciprocating saw blade parallel to the articular space was used to mobilize the tibial component. A pointed blunt impactor was inserted through a metaphyseal hole created distal to the implant to hammer the component. Once the complete removal of the implant was achieved, debridement of the epiphyseal bone and femoral and tibial canals was performed until bleeding cancellous bone was obtained, ensuring adequate penetration of postoperative antibiotic therapy. A sample of tibial and femoral cancellous bone was sent for culture examination. Once the debridement was complete, low-pressure irrigation preceded the implantation of the dynamic spacer. Careful irrigation of the soft tissues, bone, and femoral and tibial canals was performed with at least 5 L of saline solution. The residual bone was evaluated according to the Anderson Orthopedic Research Institute (AORI) classification [14], which was useful for the eventual second stage and definitive implant. Femoral varus/valgus deformities associated with bone loss were addressed using hand-made antibiotic-loaded cement augments applied to distal or posterior femoral condyles, also during the implantation phase of the mHAS. An ultracongruent/medial pivot trial liner was placed on the tibia to restore the tibial plane, choosing the correct size and thickness based on the measurements of the removed components. Subsequently, a cruciate retaining (CR) femoral component trial was placed, and a check was performed looking for rotational and axial deformities, the site for cemented augments, checking for the extension and flexion gaps, range of motion, and patella height. Once the thickness, size of liner, and femoral component were chosen, the definitive components were opened. A partially threaded cannulated screw of 6.5 mm in diameter or a Steinmann pin in larger tibial defects was inserted into the liner, replicating the function of a tibial stem in a distal to proximal direction, before being cemented on the tibial plateau, as shown in Figure 1.

**Figure 2:**
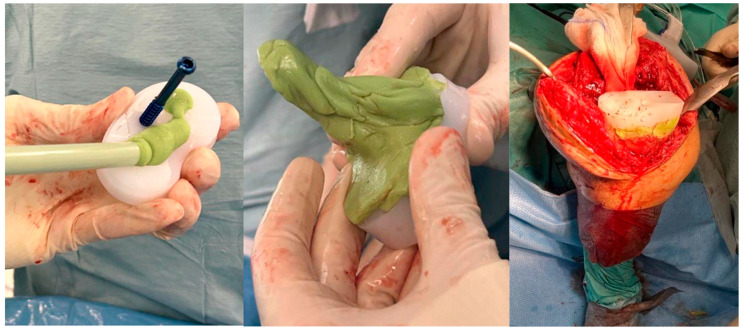
Cementation and implantation of the tibial trial liner. The inferior surface of the polyethylene liner and the partially threaded screw are fully cemented to ensure stable fixation and to fill bone gaps or defects.

A first cementation was performed, and the liner with the screw was fixed with antibiotic-loaded cement to fill the bone gap/defects. Once the liner was fixed, a second cementation was performed, and the femoral component was cemented, balancing the knee in flexion as well as in extension, as shown in Figure 2. The antibiotic-loaded cement used during the procedure contained gentamicin.

**Figure 3:**
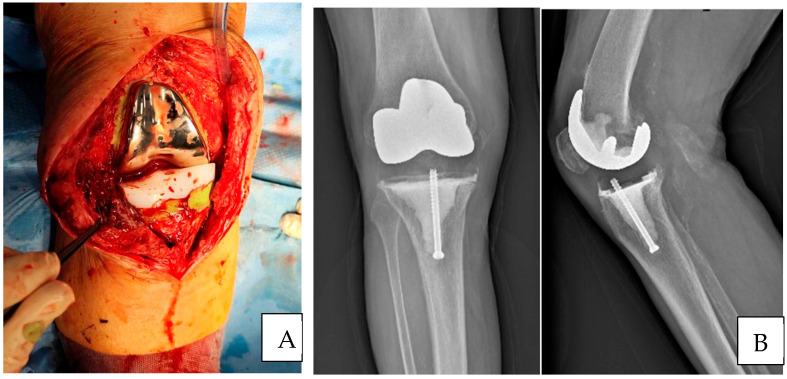
Final implantation and postoperative radiograph. (**A**) During cement fixation, the lower limb should be maintained in extension with constant traction to prevent the development of varus/valgus deformities or soft tissue shortening caused by contact between the femoral shield and the polyethylene insert. (**B**) The anteroposterior and lateral postoperative radiographs show a stable implant with adequate filling of bone defects through cementation.

During the cement fixation period, it was necessary to keep the lower limb extended with constant traction applied to prevent the development of varus/valgus deformity or shortening of the soft tissue due to contact between the femoral shield and polyethylene (Figure 3).

Primary wound closure was preferred when possible. Alternatively, an ortho-plastic approach may be used to ensure complete separation between the implanted components and the external environment, with the use of grafts or muscle flaps such as the medial gastrocnemius.

## 3. Results

Nine patients were enrolled in the study with a mean age of 74 years (range: 59–86, SD: 9.1), including four females and five males, at a mean follow-up of 8.12 months (range: 3–13). The mean surgical time was 118.5 min (range: 60–150, SD: 30.2 min), as shown in Table 1.

The bacterial pathogens identified in intraoperative culture were as follows: five cases of methicillin-sensitive *Staphylococcus aureus* (MSSA), while the remaining cases involved *Streptococcus agalactiae*, *Streptococcus anginosus*, *Corynebacterium acnes*, and methicillin-resistant *Staphylococcus epidermidis* (MRSE), as shown in Table 2.

The mean range of motion of the knee was 95 degrees (range: 80–120°, SD: 15°). The Knee Society Score (KSS) presented a mean value of 71.9 (SD: 18.11), the Oxford Knee Score (OKS) showed a mean value of 30.8 (SD: 8.5), and the EuroQol-5 Dimension-5 Level Visual Analogue Scale (EQ-5D-5L VAS) scores showed a mean value of 61, demonstrating a good quality of life among the participants. No patients experienced intraoperative or postoperative complications. At the latest radiographic evaluation, no signs of spacer mobilization were observed. At the end of follow-up, four patients, at a mean of 5 months (range: 4–7 months) after the initial surgery, with complete resolution of the infectious process, underwent removal of the Hofmann spacer and implantation of a revision knee prosthesis. In four cases, the Hofmann spacer is currently being maintained. Furthermore, infection eradication was confirmed through clinical evaluations and laboratory tests. In one case, due to persistent infection in a patient previously reimplanted, a decision was made to remove the spacer and perform knee arthrodesis using a circular external fixator. In this case, intraoperative cultures were positive for *Staphylococcus aureus*. See Table 3 and Table 4.

## 4. Discussion

The two-stage approach is widely accepted as the “gold standard” for treatment of Chronic PJI, with an infection eradication rate between 83% and 91% [15,16]. However, this approach is not without major morbidity, as failing patient outcomes remain poor and repeat two-stage exchanges have a survival free from revision at five years of 45% and a rate of re-infection of 42% [17]. In our 1.5-stage approach, no intraoperative or postoperative complications were observed during the follow-up period. With a mean age of 74 years, despite the small cohort, all patients achieved a stable knee with a minimum range of motion of 0–80° and were able to walk and perform daily activities at the mean follow-up of 8 months.

Belay’s 2022 retrospective review compared articulating spacer against standard 2-stage exchange arthroplasty for PJI reported an equivalent infection eradication rate and reoperation rate but a significantly higher overall cost of treatment and 90-day pain score for 2-stage [18]. The 1.5-stage cohort had a lower rate of postoperative complications compared to the 2-stage approach [19]. The results of our study show an 88.89% infection eradication rate with the 1.5-stage procedure in association with intravenous antibiotics.

In a retrospective review of eighty-eight cases by Sang Jun. Song et al. demonstrated survival rates of articulating spacers of 90.9%, 86.4% and 80.6% at 1, 2, and 5 years postoperatively, respectively. Inappropriate coronal positioning of the PE was the only significant factor affecting survival [20]. In our cohort in the last clinical and radiographic evaluation, no cases of spacer mobilization were observed. As outlined in the surgical technique, the use of a partially threaded cannulated screw ensures fixation to the remaining cancellous bone, providing stability to the implanted liner. The application of double cementation allows for correction of the varus-valgus deformity and a good balance in extension as well as in flexion during positioning of the femoral component with the polyethylene already fixed. Furthermore, the constant extension traction of the lower limb during the cement phase helps maintain the correction achieved, thus reducing the primary causes of failure, which are malalignment in the coronal plane or an unstable knee.

Prosthetic temporary spacers can be expected to survive up to 6 years in some studies, and often the decision to continue with the articulated spacer is the patient’s preference due to satisfactory spacer joint function without pain [21,22]. The high survival rate and mechanical reliability of the modified Hofmann articulated spacer have enabled the development of a tailored strategy in our Orthopedic and Trauma Center in the management of knee periprosthetic joint infections (PJI).

In elderly patients with significant comorbidities, prolonged retention of the modified Hofmann spacer has emerged as a viable definitive treatment option. In our cohort, patients retaining the spacer had a mean age of 81.75 years (range: 76–86) and comorbidities including obesity, diabetes, cardiomyopathy, and chronic obstructive pulmonary disease (COPD). These findings support the 1.5-stage approach as a favorable alternative in high-risk populations, offering effective infection eradication and satisfactory functional preservation while avoiding the surgical risks associated with reimplantation.

Conversely, in younger patients, mean age: 70.75 years (range: 64–75), with higher functional demands, the modified Hofmann spacer serves a different but equally important role. When used as part of a one-stage or delayed reimplantation strategy, it provided a high infection eradication rate of around 90%. A mean postoperative range of motion of 95° was observed, enabling return to daily activities and preserving joint function during the interval before definitive reimplantation. These findings highlight the spacer not only in infection management but also in maintaining quality of life throughout the treatment course.

The 1.5-stage approach represents a viable alternative for patients unable to undergo a conventional two-stage revision, primarily due to advanced age or comorbid conditions. According to our results, the use of an articulating spacer in this setting offers advantages such as reduced overall morbidity, functional preservation, and, in selected cases, the possibility of avoiding second-stage surgery.

However, this study has several limitations. The small sample size, drawn from a cohort treated between November 2023 and May 2025, may limit the generalizability of the findings. As a retrospective study, it is subject to inherent biases related to patient selection and data collection. The AORI bone loss classification was assessed for all patients as part of the surgical procedure. It was not included in the analysis, as it was not considered essential for the primary objectives of this study. To better evaluate patient-reported outcome measures (PROMs) and infection eradication rates within the 1.5-stage approach, future prospective studies should directly compare the outcomes of the modified Hofmann articulating spacer (mHAS) with those of the two-stage approach. A further limitation is that this study does not include direct comparative data between the 1.5-stage or two-stage approaches and the single-stage approach, which falls outside the scope of the present study. Future research with longer follow-up, larger sample size, and direct comparisons will be necessary to assess the efficacy, safety, and functional outcomes of these strategies more definitively.

## 5. Conclusions

The Modified Hofmann Articulated Spacer demonstrated good functional and qualitative outcomes and eradication rates in patients who underwent the first-stage revision TKA for PJI. This has led us to consider it as a potential definitive treatment option for more critical and low-demand patients and to postpone the second-stage surgery in the remaining cohort due to satisfactory spacer joint function without pain.

## Figures and Tables

**Table 1:** Demographic and clinical characteristics.

Parameter	Mean Value
Total number of patients	9
Sex (M/F)	5/4
Age (years)	74 (65–83)
Surgical time (minutes)	128.5 (SD: 30.2)
Follow-up (months)	8.12

**Table 2:** Bacterial pathogens identified in intraoperative culture.

Bacterial Pathogens	%
Methicillin-Sensitive *Staphylococcus aureus* (MSSA)	56%
*Streptococcus agalactiae*	11%
*Streptococcus anginosus* pathogen	11%
*Corynebacterium acnes*	11%
Methicillin-Resistant *Staphylococcus epidermidis* (MRSE)	11%

**Table 3:** Summary of functional and clinical outcomes.

Outcomes	Mean Value
Knee range of motion	95° (80–110°)
Knee Society Score (KSS)	71.9 (53.7–90)
Oxford Knee Score (OKS)	30.8 (22–39)

**Table 4:** Case-by-case overview of infection characteristics, antibiotic treatments, and outcomes.

Patient	Bacterial Pathogens	Antibiotic	Duration of Antibiotic Therapy	Type of Ambulation	Final Decision
1	*S. agalactiae*	Amoxicillin OP Cefazolin IV	12 weeks	Walking without crutches	Maintained Hofmann spacer
2	*S. anginosus*	Ceftriaxone IVAmoxicillin OP	6 weeks	Walking with crutches	Maintained Hofmann spacer
3	MRSE	Ceftriaxone+ Teicoplanin IV, Dalbavancin IV	3 weeks	Walking without crutches	Implantation of CCK prosthesis (2-stage)
4	MSSA	Cefazolin IV Dalbavancin IV	3 weeks	Walking with crutches	Knee arthrodesis
5	MSSA	Cefazolin IV Dalbavancin IV	3 weeks	Walking without crutches	Implantation of CCK prosthesis (2-stage)
6	MSSA	Ceftriaxone IV Doxiciclin OP	8 weeks	Walking without crutches	Maintained Hofmann spacer
7	MSSA	Ceftriaxone IV Doxiciclin OP	8 weeks	Walking with crutches	Maintained Hofmann spacer
8	MSSA	Cefazolin IV Dalbavancin IV	3 weeks	Walking without crutches	Implantation of CCK prosthesis (2-stage)
9	*C. acnes*	Vancomicin IV Amoxicillin+ Rifampicin OP	6 weeks	Walking without crutches	Implantation of CCK prosthesis (2-stage)

IV: intravenous. OP: oral administration. CCK: constrained condylar knee.

## Data Availability

The raw data supporting the conclusions of this article will be made available by the authors on request.

## Abbreviations

The following abbreviations are used in this manuscript:

mHAS	Modified Hofmann Articulated Spacer
PROMs	Patient-Reported Outcome Measures
KSS	Knee Society Score
OKS	Oxford Knee Score
TKA	Total knee arthroplasty
PJI	Periprosthetic Joint Infection
DAIR	Debridement, Antibiotic and Retention of the Implant
DAPRI	Debridement, Antibiotic Pearls and Retention of Implant
AORI	Anderson Orthopedic Research Institute
CR	Cruciate Retaining
MSSA	Methicillin-Sensitive Staphylococcus Aureus
MRSE	Methicillin-Resistant Staphylococcus Epidermidis
EQ-5D-5L VAS	EuroQol-5 Dimension-5 Level Visual Analogue Scale
CCK	constrained condylar knee
